# Response Surface Optimization of Biophotocatalytic Degradation of Industrial Wastewater for Bioenergy Recovery

**DOI:** 10.3390/bioengineering9030095

**Published:** 2022-02-26

**Authors:** Emmanuel Kweinor Tetteh, Sudesh Rathilal

**Affiliations:** Green Engineering and Sustainability Research Group, Department of Chemical Engineering, Faculty of Engineering and The Built Environment, Durban University of Technology, Durban 4001, South Africa; rathilals@dut.ac.za

**Keywords:** anaerobic digestion, bioenergy, biophotocatalysis, magnetite photocatalyst, nanotechnology, wastewater

## Abstract

The continuous combustion of fossil fuels and industrial wastewater pollution undermines global environmental and socio-economic sustainability. Addressing this necessitates a techno-scientific revolution to recover the renewable energy potential of wastewater towards a circular economy. Herein, a developed biophotocatalytic (BP) system was examined with an engineered Fe-TiO_2_ to ascertain its degradability efficiency and biogas production from industrial wastewater. The response surface methodology (RSM) based on a modified Box-Behnken designed experiment was used to optimize and maximize the BP system’s desirability. The parameters investigated included catalyst dosage of 2–6 g and hydraulic retention time (HRT) of 1–31 d at a constant temperature of 37.5 °C and organic loading rate of 2.38 kgCOD/Ld. The modified RSM-BBD predicted 100% desirability at an optimal catalyst load of 4 g and HRT of 21 d. This represented 267 mL/d of biogas and >98% COD, color, and turbidity removal. The experimental validity was in good agreement with the model predicted results at a high regression (R^2^ > 0.98) and 95% confidence level. This finding provides an insight into RSM modeling and optimization with the potential of integrating the BP system into wastewater settings for the treatment of industrial wastewater and biogas production.

## 1. Introduction

Wastewater treatment concurrently with biogas production via the anaerobic digestion (AD) process has been a universally adaptable technology [1]. However, environmental pollution, water scarcity, food, and energy insecurity have become pressing matters for sustainable development in the twenty-first century [2,3]. Also, greenhouse gas emissions (especially CO_2_) being associated with global warming, and fossil fuel combustion, undermine a sustainable environment [4]. Herein, wastewater treatment is envisioned as a renewable energy source that can be used for biogas production as alternative sources of energy [3,5]. Thus, reclaiming wastewater for reuse and biogas production (biogas can be purified and used as an automotive fuel) can ease poor country’s standard of living as far as water and energy are concerned [2].

Notwithstanding, treating industrial effluent has become extremely relevant as emerging contaminants (pharmaceuticals, pesticides, nanomaterials, etc.) and toxic chemicals originating from households and industries are posing great threats to human health and the ecosystem when discharged into the environment without proper treatment [6,7]. So, mitigating environmental pollution and its consequences warrant degradation of the high organic constituents of industrial effluents. 

Conventionally, treatment of water and wastewater involves the use of physical, chemical, and biological methods [7,8]. The biological treatment involves bacterial and fungal biosorption, anoxic and anaerobic/aerobic processes [2,7]. Membrane filtration, coagulation-flocculation, flotation, precipitation, ion exchange, adsorption, ultrasonic mineralization, ion-pair extraction, and electrolysis [6,9,10] are some of the physico-chemical treatments. Moreover, these technologies generate by-products during treatment that come with additional treatment costs [9,10]. Thus, it becomes very critical to design and select cost-effective technologies as an option in the water and wastewater treatment settings.

Currently, advanced oxidation processes (AOPs) are being developed to degrade nonbiodegradable contaminants [11]. Heterogeneous photocatalysis is a unique AOP that uses photocatalysts like TiO_2_ and UV light to break down pollutants into inoffensive end products like CO_2_, H_2_, and mineral acids [11,12]. The availability, low cost, and photochemical stability of TiO_2_ makes it a preferred photocatalytic degradation catalyst for organic contaminants [12]. To stimulate oxidation-reduction potential, photocatalytic reactions use photons with energy larger than the bandgap of a semiconductor, usually TiO_2_ [13]. Several researchers have investigated the photocatalytic treatment of hazardous substances in untreated wastewater [7,14,15,16]. Baseline pollutant concentration, photocatalyst concentration, and pH are among the key parameters that can influence photocatalytic activity [13]. Other parameters including irradiation time, light intensity, light wavelength, catalyst type and temperature can also affect the degradation routes [17]. Similarly, AD processes are influenced by several factors which includes carbon/nitrogen (C/N) ratio, temperature (mesophilic and thermophilic), organic loading rate (OLR), hydraulic retention time (HRT), and pH [1]. This makes it difficult to assess the relative importance of more than a few influencing variables, especially when they have a multifunctional effect on the outputs.

To assess the influence of operating parameters on photocatalytic process efficiency, current research has relied on a conventional one-factor-at-time (OFAT) experimental technique [18,19]. While OFAT techniques are labor-intensive, they do not depict multiple variable interaction effects. Conversely, fewer runs are needed when using response surface methodology (RSM) [20]. Box Behnken (BB), Central Composite Design (CCD), Central Composite Face centered (CCF), and full factorial are some of the most frequent design matrices used in the RSM technique. [18,19]. Environmental remediation studies have shown the great potential of using BBD and CCF for experimental design and optimization [17,18,19,20]. This is due to their ability to obtain more information from just a few numbers of their experimental matrix. Generally, using RSM in process optimization involves the following steps: (a) identification of response/s; (b) screening of multivariates according to the design of experiment; (c) building of an empirical response surface model; (d) and the application of various response optimizations through mathematical modeling [17,20]. 

**Figure 1:**
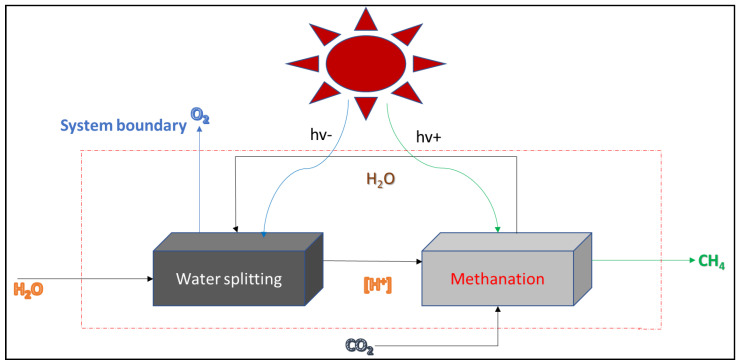
Schematic diagram of light-driven water-splitting with methanation CO_2_ reduction.

In essence, AD (kinetically slow) and AOP (energy-intensive) processes are very complex, with limited knowledge of their integrated system (AD-AOP) [21]. Therefore, developing the biophotocatalytic (BP) system as an alternative technology to the AD process comes in handy. Herein, the CO_2_ methanation mechanism was carried out by incorporating Fe-TiO_2_ into the BP system and was optimized via RSM to maximize its efficacy. Figure 1 presents the light-driven methanation reaction scheme with the dynamic state of water-splitting and CO_2_ reduction steps. Ideally, the reactant and its intermediate products emulate natural photosynthesis [22]. Similarly, the presence of the photocatalysts being energy-driven for the initial excitation of its electrons via light absorption also promotes hydro generation potential [21]. However, improper catalyst loading and operating conditions can limit microbial activity and biogas production, yielding poor methane quality. Furthermore, the development of the BP system is still underway, which warrants process modeling, optimization, and control to maximize the process efficiency, as well as support the lab-scale design of a pilot plant. Herein, this study employed experimental data obtained from a modified RSM- BBD matrix with input factors at three levels (−1, 0, +1) with two center points at a constant temperature of 37.5 °C and an organic loading rate of 2.38 kgCOD/Ld. This was aimed at investigating the relationship between the input variable (catalyst dosage and HRT) and the design outputs (biogas, COD, color, and turbidity) via the modified RSM-BBD. Additionally, the analysis of variance (ANOVA) was employed statistically to ascertain the significance of the response models, whereas numerical optimization was used to optimize and maximize the system desirability for degradation of the organics (COD) for the biogas production. 

## 2. Materials and Methods

### 2.1. Wastewater and Activated Sludge 

The raw wastewater and sludge (anaerobic digested) were obtained from the eThekwini municipal wastewater treatment plant in the KwaZulu-Natal province, South Africa by observing the standard protocols for wastewater sample collection and characterization [23]. Table 1 presents the wastewater characteristics distribution from the biofiltration stream of the wastewater treatment plant.

### 2.2. Magnetised Photocatalyst (Fe-TiO_2_)

A laboratory-based magnetite-titania photocatalyst (Fe-TiO_2_) synthesized via the co-precipitation technique was employed [24], which had a specified BET surface area of 62.73 m^2^/g, pore volume of 0.017 cm^3^/g, and 1.337 nm particle size. This was analyzed with Brunauer–Emmett–Teller theory technique equipment (Micromeritics, TriStar II Plus, Norcross, GA, USA).

### 2.3. Experimental Procedure 

**Figure 2:**
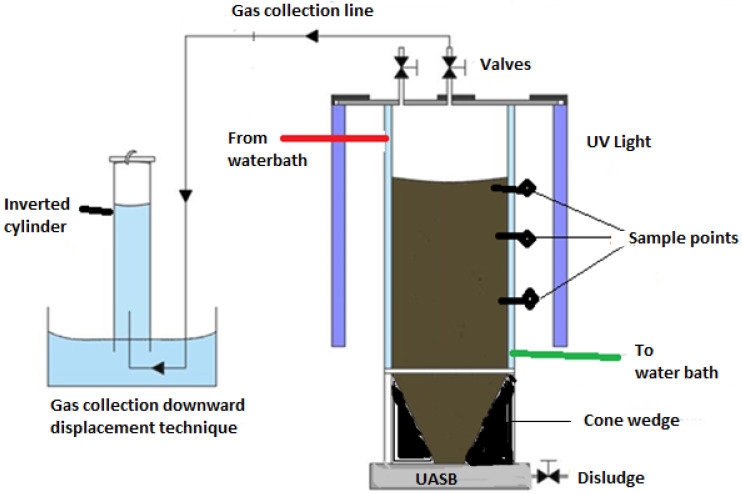
Schematic presentation of the biophotocatalytic system.

The BP system constituted an upflow anaerobic sludge blanket (UASB) reactor coupled with UV-light bulbs (T8 blacklight—blue tube, 365 nm, 18 W, Philips, The Netherlands) as depicted in Figure 2. The BP was operated in batch mode at a working volume of 8 L with a headspace of 2 L at a temperature of 37.5 °C. The effects of catalyst load and hydraulic retention time (HRT) on bio-photodegradation of the wastewater with response biogas production and water quality (COD, color, and turbidity) improvement were studied. The daily monitoring of the biogas produced was obtained via the downward displacement technique as shown in Figure 2, by reading the level of the water displaced by the measuring cylinder. The removal percentage (%R) was evaluated using Equation (1).

(1) $$Reactor\;efficiency\;\left(\%R\right)=\left(\frac{C_{i}-C_{f}}{C_{i}}\right)\times 100$$

where, $C_{i}$ = Substrate influent and $C_{f}$ = Substrate effluent. 

### 2.4. Experimental Design and Modelling 

The Design Expert software (version13.0.7) was employed for experimental design, analysis of variance (ANOVA), regression analysis (R^2^), and optimization of the process variables of the BP system. The response surface interaction between the catalyst dosage and HRT was investigated using a modified RSM-BBD method. Table 2 depicts the input variable levels considered based on the result of our previous work and other reported literature [17,18,19]. To avoid systematic mistakes, the experiment was carried out randomly with three levels, four duplicates, and one center point [25]. The response data (biogas, COD, color, and turbidity) obtained were used to develop a mathematical model that best correlates the input variables in the form of a quadratic equation (2). The model’s acceptance was also determined by the regression coefficient (R^2^) and ANOVA *p*-value.

(2) $$Y=\beta_{0}+\overset{n}{\underset{i=1}{\sum }}\beta_{i}X_{i}+\overset{n}{\underset{i=1}{\sum }}\beta_{ii}X_{ii}^{2}+\overset{n}{\underset{i<j}{\sum }}\beta_{ij}\;X_{i}X_{j}+\varepsilon$$

where the linear parameters Y, $\beta_{0}$, $\beta_{i}$, $X_{i}$ and ε represents the response, constant term, coefficient, factor, and the residual from the treatment, respectively. In addition, the quadratic terms $X_{i}$ and $X_{j}$ represents the factors, $\beta_{ii}$ and $\beta_{ij}$ represents the coefficients of the quadratic and the interaction parameters, respectively.

## 3. Results and Discussion 

**Figure 3:**
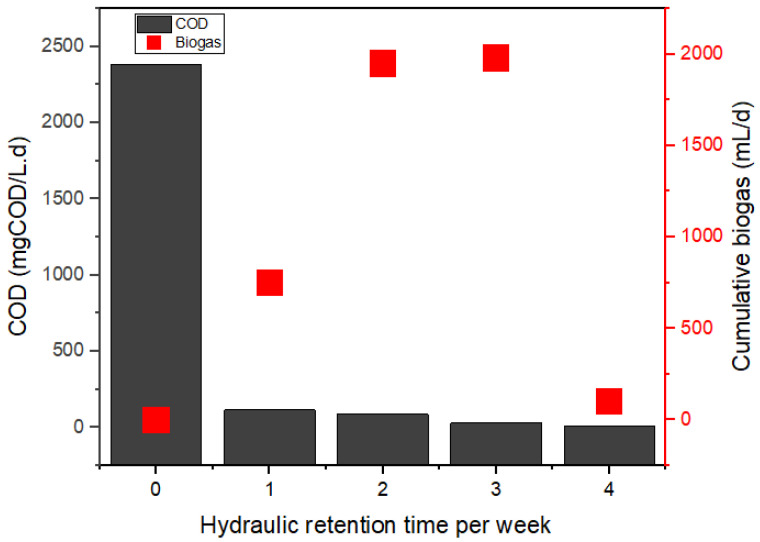
Weekly reduction of the COD and biogas produced by the BP system.

**Figure 4:**
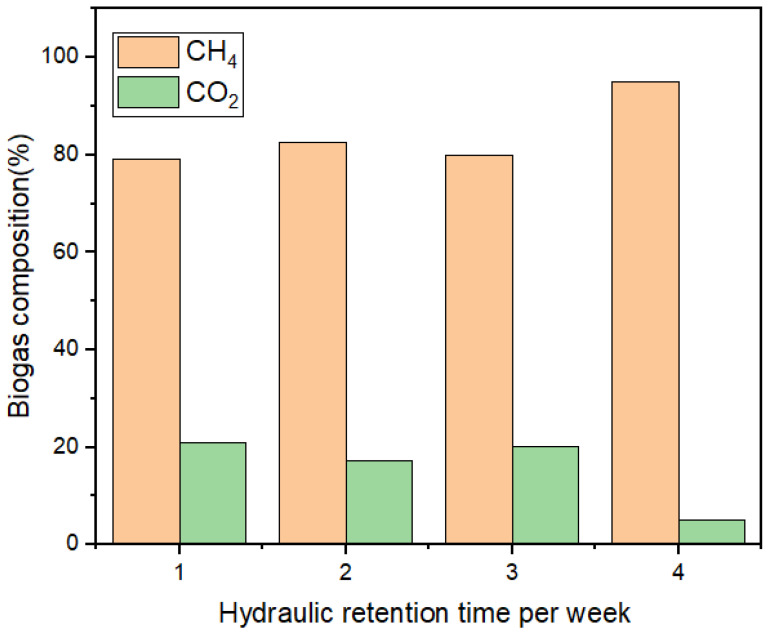
Weekly biogas composition of the BP system.

In principle, photoexcited catalysts can reduce CO_2_ to CH_4_ or split water molecules (H^+^; O^2−^) via light-induced redox reactions [22,26]. As a result (Figure 1), the H_2_ produced during the exergonic metabolism is subsequently utilized by the methanogens to enhance the CO_2_ reduction reaction into methane [1,5,27,28]. Results of the characterized municipality wastewater showed a high organic load (2380 ± 14 mgCOD/L) with a VS/TS ratio of 0.75 (Table 1). The VS/TS ratio > 0.5 proves the wastewater used was biodegradable [11,22]. Figure 3 shows the weekly monitoring of the BP system operated under anaerobic conditions, whereby the degraded organic content (2380 > 115 > 87 > 30 > 12 mgCOD/L) increased biogas production. The cumulative biogas recorded for week 1 to week 4, were 750, 1950, 1980, and 100 mL/d, respectively as depicted in Figure 3. Also, the activated photons of the Fe-TiO_2_ catalyzed the microbes to enhance the degradability of the wastewater organic content. Figure 4 shows the increased methane level of the biogas produced recorded for week 1 to week 4, respectively, like 79%, 83%, 80%, and 95%, with the rest as CO_2_ composition. From our preliminary studies, there was the need to investigate the effect of the HRT and catalyst load on the BP system efficiency. Therefore, understanding their individual or interaction effects on the BP system led to the RSM studies at a constant temperature of 37.5 °C, and an organic loading rate of 2.38 kgCOD/Ld. 

### 3.1. RSM Modelling and Statistical Analysis 

According to the modified RSM-BBD matrix (Table 2), 13 randomized runs were carried out, including one center point (run 10) and four duplicates (runs 1:3; 2:9; 5:8; and 7:12) as depicted in Table 3 with additional information in Table S1. All the responses of the center point (run 10) showed little variation, indicating the consistency of the experimental runs. In all the experimental conditions, the removal efficiency of COD, color, and turbidity was observed to range from 90–98%. This degradation efficacy corresponded to biogas production of 125–335 mL/d. However, the biogas experimental results (125–335 mL/d) were estimated to deviate by 5% to that of the modified RSM-BBD model predicted results (130–327 mL/d). Subsequently, the significance of the factors was determined by modeling the experimental data as a function of the individual response (Table 3). This resulted in a reduced quadratic model with the Design Expert software that provided equations (3–10) in their coded and actual factors for the distinctive responses. The linear (A, B), interaction (AB), and quadratic (A^2^, B^2^) terms represent the hierarchal form of the model, which combined the forward and backward regression option [29]. This regression option augments and eliminates the insignificant variables that do not meet or fall short of the model levels required (*p* < 0.05) [19,29]. The positive and negative coefficients in the predicted models (3–10) denote energetic and antagonistic effects of the process variables, respectively. In essence, the positive coefficient suggests that the response will be favored by an increase in such variable interaction. Conversely, the negative signs implies the response efficiency will decrease with an increase in those variables [29].

(3) $${\mathrm{Biogas}}_{\mathrm{coded}}\left(Y_{1}\right)=267+86.37A-3B-12.25\mathrm{AB}-50A^{2}+8.75B^{2}$$

(4) $$\begin{array}{cc} {\mathrm{Biogas}}_{\mathrm{actual}\;}\left(Y_{1}\right) & =-118.73+149.72\mathrm{Catalyst}+0.189\mathrm{HRT}-0.41\mathrm{Catalyst}\\ & *\mathrm{HRT}-12.5{\mathrm{Catalyst}}^{2}-0.03{\mathrm{HRT}}^{2} \end{array}$$

(5) $${\mathrm{COD}}_{\mathrm{coded}}\left(Y_{2}\right)=97.57+0.75A+1.13B-0.24\mathrm{AB}-1.96A^{2}-1.71B^{2}$$

(6) $$\begin{array}{cc} {\mathrm{COD}}_{\mathrm{actual}\;}\left(Y_{2}\right) & =84.53+4.44\mathrm{Catalyst}+0.35\mathrm{HRT}-0.0083\mathrm{Catalyst}*\mathrm{HRT}\\ & -0.49{\mathrm{Catalyst}}^{2}-0.03{\mathrm{HRT}}^{2} \end{array}$$

(7) $${\mathrm{Color}\;}_{\mathrm{coded}}\left(Y_{3}\right)=97.86+1.5A+0.99B-0.75\mathrm{AB}-2.07A^{2}-1.57B^{2}$$

(8) $$\begin{array}{cc} {\mathrm{Color}}_{\mathrm{actual}\;}\left(Y_{3}\right) & =82.12+5.29\mathrm{Catalyst}-0.39\mathrm{HRT}-0.0249\mathrm{Catalyst}*\mathrm{HRT}\\ & -0.518{\mathrm{Catalyst}}^{2}-0.00698{\mathrm{HRT}}^{2} \end{array}$$

(9) $${\mathrm{Turbidity}}_{\mathrm{coded}}\left(Y_{4}\right)=98.57+0.8A+0.49B-0.37\mathrm{AB}-1.03A^{2}-0.79B^{2}$$

(10) $$\begin{array}{cc} {\mathrm{Turbidity}}_{\mathrm{actual}\;}\left(Y_{4}\right) & =90.61+2.67\mathrm{Catalyst}+0.195\mathrm{HRT}-0.0125\mathrm{Catalyst}*\mathrm{HRT}\\ & -0.258{\mathrm{Catalyst}}^{2}-0.034{\mathrm{HRT}}^{2} \end{array}$$

### 3.2. Analysis of Variance (ANOVA) 

The response data was analyzed with the Design expert software, and the derived models were then fitted using the ANOVA (Table 4). The ANOVA shows how well the quadratic models fit the experimental values, parameters including F-value, probability >F, and adequate precision, which is a measure of error, or the signal-to-noise ratio were used [19,29]. The summary of the ANOVA for the various quadratic models is depicted in Table 4. The models’ variables depict the two-factor interactions between the catalyst load (A) and the HRT (B) for the biogas (Y_1_), COD (Y_2_), Color (Y_3_), and Turbidity (Y_4_) efficiency. All the variables and their interactions were significant in the proposed models except the interaction of AB of the COD (Y_2_) quadratic model (5–6) as indicated by a probability value being more than 0.05 (Table 4). A significant interaction between AB means that the effect of each variable depends on the value of the other variable [20]. Thus, increasing the catalyst load (AD) will require a longer period to reduce its potency. Adequate precision for all the models was greater than 4, suggesting a high signal/noise ratio; hence, the models can be used to navigate the design space [29].

**Figure 5:**
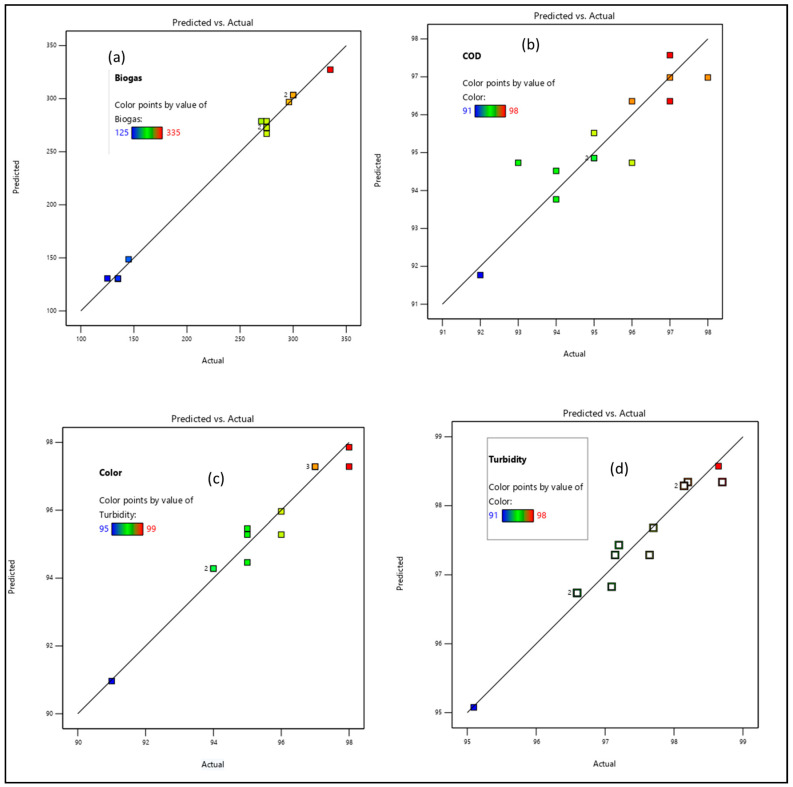
Diagnostic experimental plots against the modified RSM-BBD predicted results; (**a**) biogas, (**b**) COD, (**c**) color, and (**d**) turbidity. (The numbers on the diagonal line are the data points attained at a specific conditions of the experimental runs as inferred in Table 3).

Subsequently, the extent of correlation was estimated using experimental-predicted data interactive plots that is represented by plotting the predicted values against the experimental ones (Figure 5). Figure 5a,c,d shows a strong linear correlation between the experimental and the model predicted data with their high regression coefficients (R^2^) as depicted in Table 4. However, in Figure 5b, only a few data points were frequently scattered around the diagonal line, which could account for the on-and-off degradation of the organics (%COD removal) based on the subjected experimental conditions. The standard error (SE) for the straight line of best fit had insignificant deviation (*p* < 0.05) at a 95% confidence level. As can be inferred in Table 4, the COD (Y_2_) model regression is very low, even though all the model’s Adjusted R^2^ and Predicted R^2^ values are in reasonable agreement with a difference of less than 0.2. The standard error (SE) for the straight line of best fit shows insignificant deviation (<0.05) at 95% confidence levels.

### 3.3. One-Factor-At-Time Technique 

#### 3.3.1. Effect of Catalyst Load 

**Figure 6:**
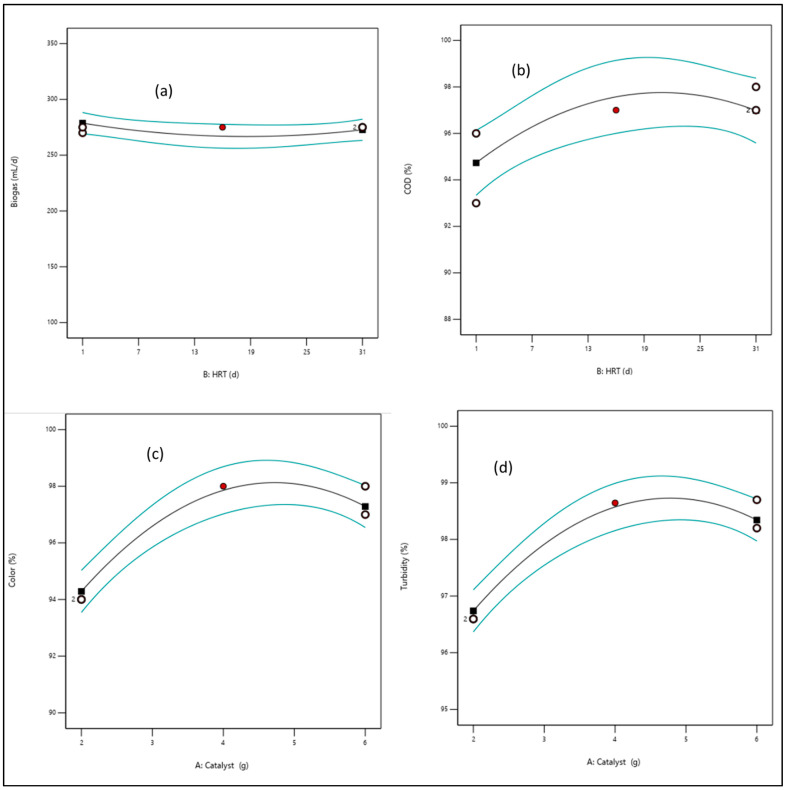
A diagnostic plot of the effect of catalyst load (2–4 g) on (**a**) biogas, (**b**) COD, (**c**) color, and (**d**) turbidity. The blue lines are the threshold or region of the response. The black is the trend line of the response. The whites and black circles are the level points of the response, whereas the red shows the high levels point.

The impact of the Fe-TiO_2_ load over the range of 2–6 g was used to study the degradability of the organic content of the wastewater into biogas. In Figure 6, it is observed that the degradation efficiency as a function of (a) biogas, (b) COD, (c) color, and (d) turbidity increased with an increase in the catalyst load. However, after the optimum regime (>4.5 g) there was a drop in the degradation efficiency. The decrease in efficiency might be due to excess hydroxyl radicals generated which agglomerated and reduced the active surface area [30]. In essence, increasing the catalyst load increased the collision frequency between the hydroxyl radicals and the organic content interspecies, which diminished the photoactivity of the system [27,30]. Also, the exponential growth of the microbial organism and their degradability activity increased biogas production [27]. This suggests that the induction of the catalyst facilitated the conductive electron interspecies transfers, which increased the microbial activity and the methanogenic activity [13,30,31].

#### 3.3.2. Effect of Hydraulic Retention Time (HRT)

**Figure 7:**
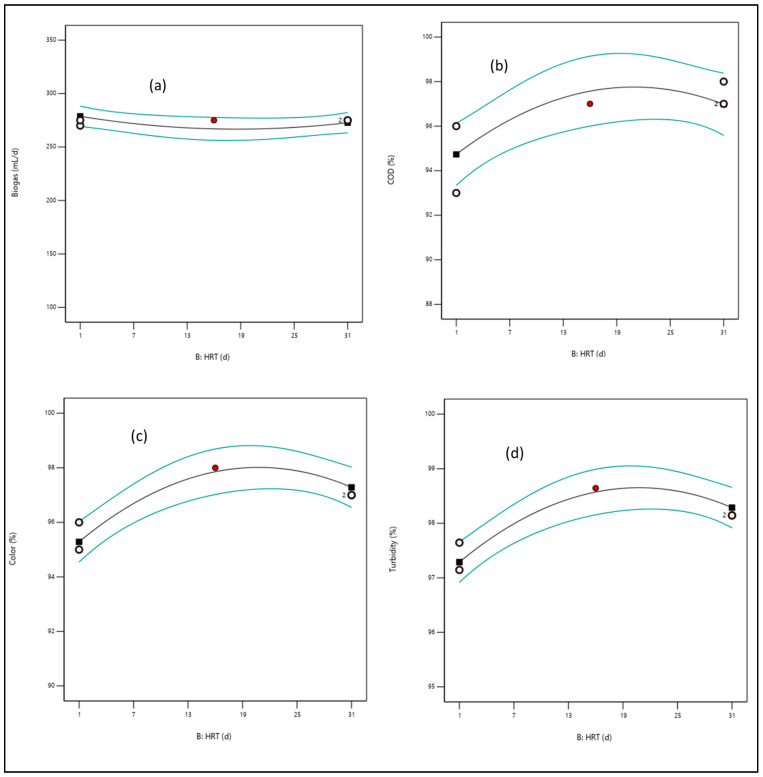
A diagnostic plot of the effect of HRT (1–31 d) on (**a**) biogas, (**b**) COD, (**c**) color, and (**d**) turbidity. The blue lines are the threshold or region of the response. The black is the trend line of the response. The whites and black circles are the level points of the response, whereas the red shows the high levels point.

The HRT investigated from 1–31 days had a significant effect on the digester performance as it facilitated microbial activity to the organic interspecies contact time, which increased its digestibility. Figure 7 shows an initial increase in degradation (COD, color, and turbidity) during the first 5 to 15 days followed by a decline (15–31 days), whereas a fairly constant rate for biogas production (Figure 7a) was observed. This indicates that a longer HRT is required for the methanogens to digest the organic component and convert it to biogas [32]. In addition, the digestion process will slow down if volatile fatty acids accumulate due to acidic bacteria being dominant with less organic content [26,30,33].

### 3.4. Response Surface Interaction Plots 

**Figure 8:**
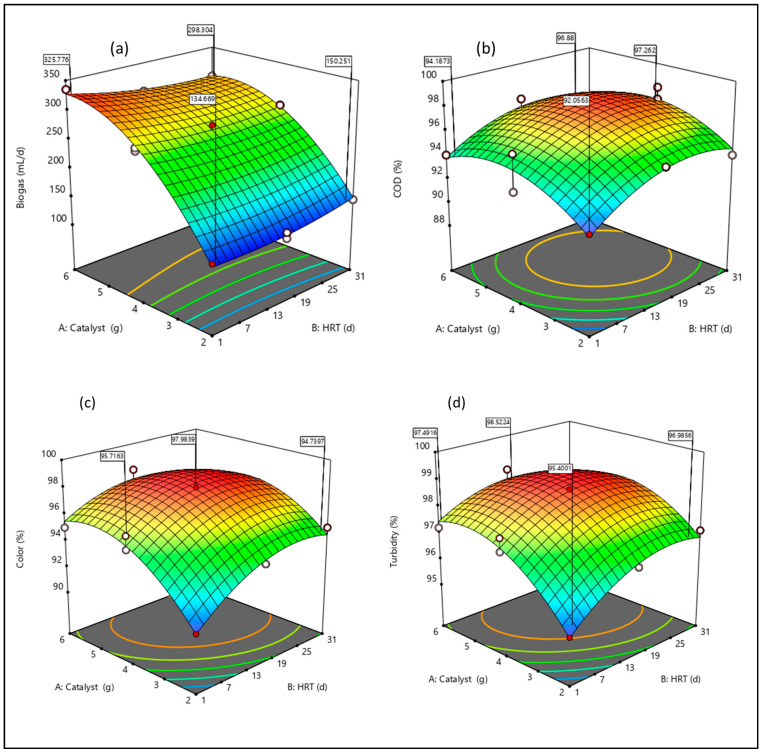
Three-dimensional (3D) plots of (**a**) biogas, (**b**) COD, (**c**) color and (**d**) turbidity.

The modified RSM-BBD was used to illustrate the interactive impact of the factors on the response. Figure 8 shows the presence of interactions between the factors, catalyst load, and HRT (AB) on (a) biogas (b) COD, (c) color, and (d) turbidity. The graphical representation of the three-dimensional (3D) surface plots of the response models was selected based on the influential factors and their interaction that can be utilized to maximize the system desirability. Curvatures of a significant magnitude can be seen in the graphs (Figure 8). These curves indicate that the correlation between the factors (AB) and the response (biogas, COD, color, and turbidity) was well fitted on a quadratic function (3–10). This elucidates Figure 8 as the degradation efficiency increased to the maximum with an increase in HRT; likewise, with the catalyst load (Figure 6). However, the curvature (Figure 8) highlights an increase in catalyst with dosage from 2 to 4 g and thereafter decreased with a further increase in catalyst load (5–6 g). The trend (Figure 8), observed at higher dosages of the catalyst, can be attributed to particle re-stabilization [30,33]. In addition, an overdose and excess of hydroxyl radicals can also aggregate to decrease the active surface area [30]. In essence, at the stage of re-stabilization, there is a charge reverse between the interspecies [26,30,33]. As illustrated (Figure 8) with an arc-line, the optimum regime can be observed within the HRT of 15–25 days. This validates the positive sign of the response quadratic models (3–10) as reported earlier, which had significant impact on the system predictability (Figure 5). 

### 3.5. Response Optimization and Confirmation Test 

**Figure 9:**
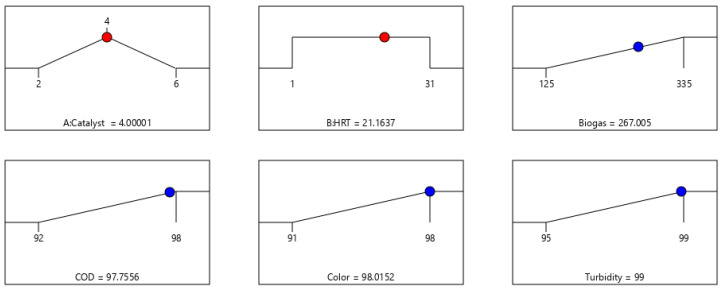
Selected numerical optimized condition ramp plots.

The numerical optimization technique was employed to maximize the responses (biogas, COD, color, and turbidity) and determine the optimal conditions (with respect to the experimental runs) using their respective quadratic equations (3–10). Also, the comparative evaluation of the experimental and model predicted responses at each level of the experimental conditions (Table 3) showed considerable correlation with the two input variables (A and B) as a constraint. The goal for the optimization was defined as a function of the input variables set within the range of their levels such as low (−1) and high (+1) to maximize the desirability of the responses. The optimal solution selected out of 78 solutions (Table S1) to maximize the responses is presented in Figure 9 with 100% desirability. As it can be inferred from the ramp plot (Figure 9), 267 mL/d of biogas, 97.75% COD, 98% color, and 99% turbidity removal were attained at 4 g catalyst load and HRT of 21 d. The selected optimal conditions validated and confirmed experimentally as presented in Table 5 were in good agreement with the predicted values. This suggests the model’s predictability was consistent (*p* < 0.05) at 95% confidence levels with a small standard error (SE) and standard deviation (STD) (Table 5). Also, the results obtained were compared with other studies (Table 6), which showed the appreciable efficacy of the BP system for wastewater treatment and biogas production. 

## 4. Conclusions

In this study, biogas, and decontamination (COD, color, and turbidity removal) efficiency of biophotocatalytic degradation of municipality wastewater was studied. The lab-scale biophotocatalytic (BP) system operated in batch mode was augmented with Fe-TiO_2_ and optimized to treat the wastewater. The results demonstrated both the HRT and catalyst load had positive effects on the BP system efficacy. At an optimal catalyst load of 4 g and HRT of 21 d, the modified RSM-BBD model predicted results were validated experimentally, and 267 mL/d of biogas and >98% COD, color and turbidity removal efficiency were attained. This infers 100% desirability with a high coefficient of regression of >0.98 at 95% confidence. The analysis of variance (ANOVA) suggested the modified RSM-BBD response models developed were significant with high precision, as the predicted results and the experimental results were in good agreement. This study demonstrates the modified RSM-BBD’s usefulness and reliability in modeling, optimizing, and monitoring the effectiveness of the BP systems for wastewater treatment and biogas production.

## Figures and Tables

**Table 1:** Wastewater characteristics and analytical technique.

Water Quality	Value	Analytical Units
pH	7.42 ± 2.3	Hanna pH/EC/TDS Tester (H198130)
Temperature (°C)	26.42 ± 3.6	Hanna pH/EC/TDS Tester (H198130)
Color (abs 465 nm, Pt. Co)	570.23 ± 12	HACH Spectrophotometer (DR3900)
Turbidity (NTU)	732.32 ± 14	Turbidity meter (HACH 2100N)
Chemical oxygen demand (mg COD/L)	2380.32 ± 14	HACH Spectrophotometer (DR3900)
Ammonia (mg NH_3_/L)	0.74 ± 0.4	HACH Spectrophotometer (DR3900)
Total Kjeldahl nitrogen (mg TKN/L)	30.52 ± 1.4	HACH Spectrophotometer (DR3900)
Nitrate (mg NO_3_/L)	0.64 ± 0.5	HACH Spectrophotometer (DR3900)
Total nitrogen (mg TN/L)	31.88 ± 7.8	HACH Spectrophotometer (DR3900)
Total suspended solids (mgTS/L)	304.53 ± 15.6	Analytical balance (HCB602H 22 ADAM)
Volatile solids (mg VS/L)	229.52 ± 25	Analytical balance (HCB602H 22 ADAM)
Ratio (%VS/TS)	75.37	

**Table 2:** Box- Benhken design matrix.

Symbol	Factor Name	Unit	Type	Low	Middle	High
Coded level				−1	0	1
A	Catalyst load	g	Factor	2	4	6
B	HRT	d	Factor	1	16	31

**Table 3:** Results of a modified RSM-BBD experiment and model predictions.

	Factor 1	Factor 2	Experimental Results				RSM-BBD Model Predicted Results			
Run	A:Catalyst Load (g)	B:HRT (d)	Biogas (mL/d)	COD (%)	Color (%)	Turbidity (%)	Biogas (mL/d)	COD (%)	Color (%)	Turbidity (%)
1	2	16	135	95	94	96.6	130.6	94.9	94.3	96.7
2	6	16	300	96	97	98.2	303.4	96.4	97.3	98.3
3	2	16	125	95	94	96.6	130.6	94.9	94.3	96.7
4	6	1	335	94	95	97.2	327.4	93.8	95.5	97.4
5	4	31	275	97	97	98.1	272.8	97.0	97.3	98.3
6	2	31	145	94	95	97.1	148.6	94.5	94.5	96.8
7	4	1	275	96	96	97.6	278.8	94.7	95.3	97.3
8	4	31	275	98	97	98.1	272.8	97.0	97.3	98.3
9	6	16	300	97	98	98.7	303.4	96.4	97.3	98.3
10	4	16	275	97	98	98.6	267.0	96.6	96.9	97.6
11	6	31	296	95	96	97.7	296.9	95.5	96.0	97.7
12	4	1	270	93	95	97.1	278.8	94.7	95.3	97.3
13	2	1	135	92	91	95.1	130.1	91.8	91.0	95.1

**Table 4:** A modified RSM-BBD. model analysis of variance (ANOVA).

Response	Source	Sum of Squares	df	F-Value	*p*-Value	R^2^	Adeq Precision
Biogas (Y_1_)	Model	69,092.95	5	289.5	<0.0001	0.9952	42.025
	A-Catalyst	59,685.12	1	1250.42	<0.0001		
	B-HRT	72	1	1.51	0.2591		
	AB	600.25	1	12.58	0.0094		
	A^2^	7000	1	146.65	<0.0001		
	B^2^	214.38	1	4.49	0.0718		
	Residual	334.13	7				
COD(Y_2_)	Model	29.57	5	20.48	0.0005	0.9360	16.0298
	A-Catalyst	4.5	1	17.65	0.0040		
	B-HRT	10.12	1	25.00	0.0016		
	AB	0.25	1	0.6449	0.4483		
	A^2^	10.8	1	49.41	0.0002		
	B^2^	8.23	1	25.78	0.0014		
	Residual	7.2	7				
Color (Y_3_)	Model	43.04	5	29.6	0.0001	0.9548	18.8142
	A-Catalyst	18	1	61.89	0.0001		
	B-HRT	8	1	27.51	0.0012		
	AB	2.25	1	7.74	0.0272		
	A^2^	12.01	1	41.31	0.0004		
	B^2^	6.91	1	23.78	0.0018		
	Residual	2.04	7				
Turbidity (Y_4_)	Model	11.4	5	31.36	0.0001	0.9573	19.0872
	A-Catalyst	5.15	1	70.86	<0.0001		
	B-HRT	2	1	27.51	0.0012		
	AB	0.5625	1	7.74	0.0272		
	A^2^	2.99	1	41.11	0.0004		
	B^2^	1.73	1	23.78	0.0018		
	Residual	0.5089	7				

**Table 5:** Modified RSM-BBD optimum conditions experimental validation.

Response	Predicted	Observed	* Std Dev	* SE Mean
Biogas (mL/d)	267	250	6.95	4.53
COD (%)	98	95	1.01	0.67
Color (%)	98	96	0.53	0.38
Turbidity (%)	99	97	1.87	1.67

* Std Dev—Standard deviation, SE—standard error.

**Table 6:** Comparing previous and current studies.

Waste Type	Process	Operating Condition	Efficiency	Reference
Blast furnace sludge (BFS) with a Fe-rich residue, as a catalyst	A Laboratory scale differential reactor	Temperature of 300–350 °C, 1 atm, and variable partial pressures of H_2_ (10–50 kPa) and CO (0.25–3.0 kPa)	The methane production and selectivity achieved were 2.63 μmolCH_4_/gcat/min and 49.5%	[22]
Municipality wastewater seeded with 2 g of Fe_2_O_4_-TiO_2_ MNPs	Biochemical Methane Potential (BMP) Test	Temperature 40 °C for 30 days	biogas production (400 mL/day) and methane yield (100% CH_4_)	[24]
Municipality wastewater	Biochemical Methane Potential (BMP) Test	Temperature 40 °C for 30 days	Biogas production (350 mL/day) and methane yield (65% CH_4_).	[24]
Distillery wastewater	Integrated anaerobic -photocatalysis	Organic load rate (OLR) of 3 kg COD/m^3^.d) and hydraulic retention time (HRT) of 20 days	98% COD, 50% color, bioenergy of 180.5 kWh/m^3^	[25]
Lignocellulosic materials	Anaerobic digestion	0.252 mg of NiO–TiO_2_/g total solids (TS) and HRT of 4 days	Soluble chemical oxygen demand (COD) and 67% increase in volatile fatty acids (VFAs)	[33]
Municipality wastewater seeded with Fe-TiO_2_	Biophotocatalytic system	4 g catalyst load and HRT of 21 d	267 mL/d of biogas, 97.75% COD, 98% color and 99% turbidity	This study

## Data Availability

No new data were created or analyzed in this study. Data sharing is not applicable to this article.

## Supplementary Materials

The following supporting information can be downloaded at: https://www.mdpi.com/article/10.3390/bioengineering9030095/s1, Table S1: Modified RSM-BBD optimized conditions of the BP system.
