# Supplementary material for: Response Surface Optimization of Biophotocatalytic Degradation of Industrial Wastewater for Bioenergy Recovery
Source: Bioengineering (Basel). 2022 Feb 26;9(3):95. doi: 10.3390/bioengineering9030095 (PMC8945768; doi:10.3390/bioengineering9030095)
Supplement: Supplementary file 1 [file bioengineering-09-00095-s001.zip › bioengineering-1565307-supplementary.pdf]

Response Surface Optimization of Biophotocatalytic Degradation of Industrial Wastewater for Bioenergy Recovery

Emmanuel Kweinor Tetteh and Sudesh Rathilal

Table S1. Modified RSM-BBD optimized conditions of the BP system.

Number	Catalyst Load (g)	HRT (days)	Biogas (mL/d)	COD (%)	Color (%)	Turbidity (%)
**1**	**4**	***16***	**267**	**97.571**	**97.857**	**98.574**
2	4	*31*	272.75	96.982	97.286	98.288
3	4	*1*	278.75	94.732	95.286	97.288
4	4	*16.796*	266.865	97.626	97.906	98.598
5	4	*27.905*	270.131	97.384	97.661	98.475
6	4	*18.504*	266.743	97.711	97.98	98.635
7	4	*14.443*	267.406	97.436	97.736	98.513
8	4	*9.324*	270.069	96.731	97.101	98.195
9	4	*10.059*	269.561	96.857	97.215	98.252
10	4	*12.025*	268.409	97.153	97.482	98.386
11	4	*25.345*	268.527	97.607	97.87	98.58
12	4	*25.461*	268.589	97.599	97.863	98.576
13	4	*15.147*	267.199	97.502	97.795	98.543
14	4	*18.696*	266.743	97.718	97.986	98.638
15	4	*17.438*	266.793	97.664	97.939	98.614
16	4	*20.922*	266.958	97.756	98.016	98.653
17	4	*17.939*	266.758	97.688	97.96	98.625
18	4	*23.4*	267.65	97.709	97.968	98.629
19	4	*5.348*	273.543	95.908	96.354	97.822
20	4	*23.201*	267.576	97.716	97.975	98.633
21	4	*27.037*	269.53	97.471	97.742	98.516
22	4	*25.644*	268.688	97.586	97.85	98.57
23	4	*22.022*	267.206	97.747	98.005	98.648
24	4	*5.641*	273.245	95.977	96.417	97.853
25	4	*4.802*	274.116	95.776	96.235	97.762
26	4	*3.521*	275.551	95.449	95.938	97.614
27	4	*21.892*	267.172	97.749	98.007	98.649
28	4	*11.262*	268.821	97.045	97.384	98.337
29	4	*15.46*	267.119	97.529	97.819	98.555
30	4	*16.993*	266.84	97.638	97.916	98.603
31	4	*13.253*	267.843	97.308	97.621	98.456
32	4	*19.802*	266.802	97.746	98.01	98.65
33	4	*19.047*	266.752	97.729	97.995	98.643
34	4	*19.988*	266.821	97.749	98.012	98.651
35	4	*11.394*	268.746	97.064	97.402	98.346

36	4	*20.121*	266.836	97.751	98.013	98.652
37	4	*22.406*	267.315	97.739	97.998	98.644
38	4	*17.641*	266.777	97.674	97.948	98.619
39	4	*23.58*	267.718	97.702	97.961	98.626
40	4	*14.572*	267.365	97.449	97.748	98.519
41	4	*5.749*	273.137	96.002	96.44	97.865
42	4	*20.297*	266.859	97.753	98.015	98.652
43	4	*6.772*	272.157	96.23	96.647	97.969
44	4	*21.422*	267.059	97.754	98.013	98.652
45	4	*14.955*	267.251	97.485	97.78	98.535
46	4	*26.124*	268.961	97.55	97.816	98.553
47	4	*19.666*	266.789	97.744	98.008	98.649
48	4	*13.845*	267.612	97.374	97.681	98.486
49	4	*17.2*	266.816	97.65	97.927	98.609
50	4	*7.638*	271.392	96.411	96.811	98.051
51	4	*24.751*	268.228	97.644	97.906	98.598
52	4	*14.838*	267.285	97.474	97.77	98.53
53	4	*4.431*	274.519	95.684	96.151	97.721
54	4	*3.998*	275.002	95.574	96.051	97.67
55	4	*16.452*	266.917	97.604	97.886	98.588
56	4	*12.296*	268.274	97.189	97.514	98.402
57	4	*3.251*	275.871	95.377	95.872	97.581
58	4	*19.541*	266.779	97.741	98.006	98.648
59	4	*19.399*	266.77	97.738	98.003	98.647
60	4	*8.582*	270.623	96.596	96.978	98.134
61	4	*14.685*	267.33	97.46	97.757	98.524
62	4	*29.173*	271.114	97.237	97.523	98.407
63	4	*23.843*	267.823	97.691	97.95	98.62
64	4	*2.656*	276.594	95.214	95.724	97.507
65	4	*12.566*	268.146	97.224	97.546	98.418
66	4	*27.532*	269.866	97.423	97.697	98.494
67	4	*27.214*	269.647	97.454	97.726	98.508
68	4	*7.386*	271.608	96.36	96.765	98.027
69	4	*10.193*	269.473	96.879	97.234	98.262
70	4	*17.332*	266.802	97.658	97.934	98.612
71	4	*22.678*	267.4	97.733	97.991	98.64
72	4	*11.63*	268.612	97.098	97.432	98.361
73	3.9	*28.75*	266.849	97.257	97.523	98.404
74	3.9	*18.25*	262.395	97.661	97.897	98.591
75	3.9	*3.25*	270.908	95.324	95.76	97.522
76	4.1	*28.75*	274.445	97.311	97.61	98.452
77	4.1	*18.25*	270.849	97.732	98.036	98.666
78	4.1	*3.25*	280.586	95.42	95.973	97.634
